# Short-Term and Long-Term Mortality Risk After Preterm Birth

**DOI:** 10.1001/jamanetworkopen.2024.45871

**Published:** 2024-11-20

**Authors:** Asma M. Ahmed, Sonia M. Grandi, Eleanor Pullenayegum, Sarah D. McDonald, Marc Beltempo, Shahirose S. Premji, Jason D. Pole, Fabiana Bacchini, Prakesh S. Shah, Petros Pechlivanoglou

**Affiliations:** 1 Department of Epidemiology and Prevention, Wake Forest University School of Medicine, Winston-Salem, North Carolina; 2 Child Health Evaluative Sciences, The Hospital for Sick Children, Toronto, Ontario, Canada; 3 Division of Maternal-Fetal Medicine, Department of Obstetrics and Gynecology, McMaster University, Hamilton, Ontario, Canada; 4 Department of Pediatrics, McGill University, Montreal, Quebec, Canada; 5 School of Nursing, Faculty of Health Sciences, Queen’s University, Kingston, Ontario, Canada; 6 Centre for Health Services Research, The University of Queensland, Brisbane, Australia; 7 Canadian Premature Babies Foundation, Toronto, Ontario, Canada; 8 Department of Pediatrics, Mount Sinai Hospital, Toronto, Ontario, Canada

## Abstract

**Importance:**

Preterm birth (PTB) is a leading cause of neonatal mortality. However, evidence on mortality beyond the neonatal period is limited, especially in North America.

**Objective:**

To examine associations of PTB with all-cause and cause-specific mortality from birth through 23 to 36 years of age.

**Design, Setting, and Participants:**

This population-based matched cohort study of live births in Canada included individuals born between January 1, 1983, and December 31, 1996, and followed up until December 31, 2019. Data were analyzed from June 1, 2023, to April 30, 2024.

**Exposure:**

PTBs, between 24 and 37 weeks’ gestation (with gestational age [GA]-specific subcategories of 24 to 27 weeks, 28 to 31 weeks, 32 to 33 weeks, and 34 to 36 weeks) compared with term births (37-41 weeks’ gestation).

**Main Outcomes and Measures:**

All-cause mortality and cause-specific mortality were the main outcomes. Risk differences (RDs) and risk ratios (RRs) were estimated for all-cause mortality using log-binomial regressions and hazard ratios were estimated for cause-specific mortality using Cox proportional hazards regression models (censoring individuals who died from other causes) within prespecified age intervals (ages 0-11 months and ages 1-5, 6-12, 13-17, 18-28, and 29-36 years). Observed confounding was accounted for using coarsened exact matching on baseline characteristics.

**Results:**

Of 4 998 560 births (54.2% male), 6.9% were born preterm (with GA-specific subcategories of 0.3% born at 24-27 weeks, 0.6% at 28-31 weeks, 0.8% at 32-33 weeks, and 5.1% at 34-36 weeks). During a median of 29 years of follow-up, 72 662 individuals died (14 312 born preterm and 58 350 at term). PTB was associated with an increased risk of death in all age intervals, with the highest RDs and RRs from birth through infancy (ages 0-11 months) (RD, 2.29% [95% CI, 2.23%-2.35%]; RR, 11.61 [95% CI, 11.09-12.15]) and in early childhood (ages 1-5 years) (RD, 0.34% [95% CI, 0.31%-0.36%]; RR, 2.79 [95% CI, 2.61-2.98]) and the lowest RDs and RRs among those between ages 18 and 28 years (RD, 0.07% [95% CI, 0.04%-0.10%]; RR, 1.13 [95% CI, 1.07-1.19]). We identified increased risks of mortality associated with several causes, including respiratory, circulatory, and digestive system disorders; nervous system, endocrine, and infectious diseases; cancers; congenital malformations; and conditions originating in the perinatal period. No associations were found for external causes of deaths. Associations by GA categories suggested lower risks with higher GA.

**Conclusions and Relevance:**

The findings of this population-based matched cohort study suggest that individuals born preterm were at an increased risk of death from birth until their third and fourth decades of life, with higher risks as GA decreased. Some of these associations may have been partly due to underlying health determinants that affected PTB and mortality. These findings suggest that PTB should be recognized as a risk factor for mortality and could inform preventive strategies.

## Key Points

**Question:**

Do individuals born preterm have a higher risk of short-term and long-term mortality compared with those born at term?

**Findings:**

In this cohort study of 4 998 560 births, individuals born preterm had a higher risk of mortality from birth to age 36 years, with the highest risk observed from birth through early childhood. The risk of mortality varied by gestational age at birth and was more pronounced at lower gestational ages.

**Meaning:**

The findings of this study suggest that preterm birth is associated with increased risk of death from infancy to adulthood.

## Teaser

This cohort study of live births in Canada examines associations of preterm birth with all-cause and cause-specific mortality from birth to age 36 years.

## Introduction

Preterm birth (PTB), defined as birth before 37 weeks’ gestation, is one of the most common adverse perinatal outcomes, affecting approximately 10% of all births worldwide.^1,2^ PTB is the leading cause of neonatal mortality and the second leading cause of death globally among children younger than 5 years.^3^ Rates of PTB have been rising in many countries, attributed to increased reporting of births before 28 weeks’ gestation, physician-initiated PTB due to changes in obstetric practices, and multiple gestation pregnancies conceived using assisted reproductive techniques.^3,4^ Prematurity affects many organ systems, leaving individuals who are born preterm vulnerable to a variety of short-term and long-term morbidities across their lifespan.^5,6^ The rates of morbidity and mortality associated with PTB are inversely associated with gestational age (GA) at birth, with those born younger than 32 weeks’ gestation disproportionately accounting for about 50% of infant deaths in the US (PTBs <32 weeks’ gestation only constitute about 2% of live births).^7,8^

Although the majority of individuals born preterm survive into adulthood, growing evidence suggests that they remain at increased risk of death across their life course.^9,10,11,12,13,14,15^ Most previous population-based studies have focused on the first few years of life, supporting an increased risk of mortality after PTB.^5,8,15,16,17,18^ However, only a few studies, mostly from Europe, have examined mortality during adulthood.^9,10,11,12,13,14^ A recent systematic review showed an increased risk of death during adulthood among individuals who were born preterm (risk ratios [RRs], 1.2-1.6).^10^ Most studies included in that review, however, were conducted in a single country (Sweden). A recent study including populations from 4 Nordic countries also found an increased risk of mortality from 15 to 51 years of age associated with PTB.^13^ Therefore, there is still a need for a study from North America, given the differences between North American population characteristics and those of Nordic European countries.

To our knowledge, this is the first population-based study in North America to assess the associations between PTB and all-cause and cause-specific mortality from birth through adulthood (23-36 years of age). We used novel vital statistics data and a matched cohort design that accounted for various sociodemographic characteristics.

## Methods

### Settings

In this cohort study, we created a population-based birth cohort using vital statistics data from Statistics Canada.^19^ Statistics Canada is a government agency responsible for housing data and producing statistics to enhance understanding of Canada’s demographics, resources, economy, society, and culture.^19^ Data of live births in Canada between January 1, 1983, and December 31, 1996, were included. We excluded births with missing information on baseline covariates or missing or invalid GA (birth weight for GA *z* score >4 SD above or below the mean),^20^ births with GAs less than 24 weeks (due to the underreporting of neonatal deaths at early GA),^21,22^ and postterm births more than 41 weeks’ gestation. The cohort was followed up until December 31, 2019, resulting in a minimum of 23 years and a maximum of 36 years of follow-up. We linked the Canadian Vital Statistics—Birth database^23^ and the Canadian Vital Statistics—Death^24^ database files, which are cross-sectional administrative databases that collect information on live births and deaths from all Canadian provincial and territorial vital statistics registries. Data privacy and confidentiality were upheld throughout the process of data linking and use, with access involving use of deidentified data through a secure server provided by Statistics Canada. Approval for the study was obtained from The Hospital for Sick Children research ethics board and Statistics Canada, which waived the need for informed consent owing to the use of deidentified data. We followed the Strengthening the Reporting of Observational Studies in Epidemiology (STROBE) reporting guideline.

### PTB

Information about GA at birth was available from the Canadian Vital Statistics—Birth database and was completed by parents and health care practitioners. We defined PTB as a binary variable (<37 weeks’ vs 37-41 weeks’ gestation) and a multicategory variable (extremely preterm: 24-27 weeks’ gestation, very preterm: 28-31 weeks’ gestation, moderately preterm: 32-33 weeks’ gestation, and late preterm: 34-36 weeks’ gestation) vs term (37-41 weeks’ gestation).

### Study Outcome

Mortality data were obtained from the Canadian Vital Statistics—Death database file, which included the underlying cause and date of death. All-cause mortality was defined as death from any cause during the follow-up period. Individuals without a recorded death by the end of follow-up were assumed to be alive. For cause-specific mortality, we categorized death according to the recorded underlying cause of death based on the *International Classification of Diseases, Ninth Revision* and the *International Statistical Classification of Diseases and Related Health Problems, Tenth Revision* codes (details in eTable 1 in Supplement 1).^25,26^ These categories included respiratory system disorders; circulatory disorders; diseases of the nervous system; infectious diseases; digestive system disorders; endocrine, nutritional and metabolic diseases; cancer; external causes of mortality; mental and behavioral disorders; congenital malformation; and conditions originating in the perinatal period (eg, low birth weight or PTB, birth asphyxia, and respiratory distress syndrome and perinatal infections).

### Baseline (Matching) Covariates

We matched on the following baseline covariates: individuals’ sex (male, female), birth plurality (singleton, multiple), province of birth, birth year, parental age at birth (<20, 20-24, 25-29, 30-34, 35-39, and ≥40 years for the mother and <25, 25–29, 30–34, 35–39, and ≥40 years or missing information for the father), maternal marital status (single, married, other [included widowed, divorced, or separated], or missing information), maternal parity (0, 1, 2, 3, or ≥4 previous live births), and parental origin of birth (Canada, North America excluding Canada, Central and South America, Europe, Africa, Asia, and other [Oceania or Antarctica and adjacent islands and missing or unknown data]) based on the Statistical Classification of Countries and Areas of Interest for social statistics.^27^

### Statistical Analysis

Data were analyzed from June 1, 2023, to April 30, 2024. Coarsened exact matching was used to match individuals born preterm with those born at term, based on the aforementioned measured confounding factors .^28^ Coarsened exact matching is a matching method used to achieve balance between treatment and control groups by grouping individuals with similar covariate patterns, thereby reducing confounding bias.^28^ We categorized matching variables into broader categories (as outlined in Table 1), created multiple strata based on distinct combinations of covariate categories, and then matched individuals with identical patterns of these categories. This matching process resulted in strata with varying numbers of individuals born preterm and at term. To adjust for these differences in stratum sizes, we applied weights in all subsequent analyses, ensuring comparable covariate distributions between the groups. Strata without individuals who were born preterm or at term were excluded.^28^ Matching was repeated separately for each of the GA subcategories.

**Table 1.** Characteristics of Individuals Born at Term and Preterm in Unmatched and Matched Cohorts in Canada for Births from 1983 to 1996

Characteristics	Cohort, No. (%)^a^				
	Unmatched (N = 4 998 560)			Matched (weighted) (N = 4 350 210)	
	Term birth (n = 4 655 980)	Preterm birth (n = 342 580)	SMD	Term birth (n = 4 033 880)	Preterm birth (n = 316 330)
Sex					
Female	2 277 730 (48.9)	156 770 (45.8)	0.06	1 839 490 (45.6)	144 250 (45.6)
Male	2 378 250 (51.1)	185 810 (54.2)		2 194 390 (54.4)	172 080 (54.4)
Birth plurality					
Singleton	4 597 440 (98.7)	293 030 (85.5)	0.51	3 605 150 (89.4)	282 710 (89.4)
Multiple	58 540 (1.3)	49 550 (14.5)		428 720 (10.6)	33 620 (10.6)
Maternal parity					
0	1 999 540 (42.9)	158 450 (46.3)	0.10	1 925 140 (47.7)	150 970 (47.7)
1	1 669 660 (35.9)	108 640 (31.7)		1 289 770 (32.0)	101 140 (32.0)
2	684 860 (14.7)	48 760 (14.2)		553 680 (13.7)	43 420 (13.7)
3	203 080 (4.4)	17 090 (5.0)		175 700 (4.4)	13 780 (4.4)
≥4	98 850 (2.1)	9640 (2.8)		89 580 (2.2)	7030 (2.2)
Maternal age, y					
<20	277 470 (6.0)	26 240 (7.7)	0.11	311 660 (7.7)	24 440 (7.7)
20-24	1 029 950 (22.1)	77 760 (22.7)		930 470 (23.1)	72 970 (23.1)
25-29	1 735 490 (37.3)	116 650 (34.0)		1 404 930 (34.8)	110 170 (34.8)
30-34	1 199 010 (25.8)	85 070 (24.8)		998 630 (24.8)	78 310 (24.8)
35-39	366 590 (7.9)	31 790 (9.3)		344 870 (8.5)	27 040 (8.5)
≥40	47 480 (1.0)	5070 (1.5)		43 320 (1.1)	3400 (1.1)
Paternal age, y					
<25	553 490 (11.9)	44 730 (13.1)	0.13	528 460 (13.1)	41 440 (13.1)
25–29	1 415 490 (30.4)	97 230 (28.4)		1 175 120 (29.1)	92 150 (29.1)
30–34	1 424 850 (30.6)	94 970 (27.7)		1 139 050 (28.2)	89 320 (28.2)
35–39	652 540 (14.0)	47 760 (13.9)		546 460 (13.5)	42 850 (13.5)
≥40	267 740 (5.8)	22 800 (6.7)		237 740 (5.9)	18 640 (5.9)
Missing	341 870 (7.3)	35 090 (10.2)		407 050 (10.1)	31 920 (10.1)
Maternal place of birth					
Africa	30 370 (0.7)	2630 (0.8)	0.11	22 000 (0.5)	1730 (0.5)
Asia	214 530 (4.6)	19 170 (5.6)		210 750 (5.2)	16 530 (5.2)
Canada	3 881 490 (83.4)	274 790 (80.2)		3 324 710 (82.4)	260 720 (82.4)
Central and South America	59 070 (1.3)	5780 (1.7)		52 950 (1.3)	4150 (1.3)
Europe	233 930 (5.0)	16 550 (4.8)		170 430 (4.2)	13 370 (4.2)
North America excluding Canada	85 160 (1.8)	6370 (1.9)		57 700 (1.4)	4530 (1.4)
Other^b^	151 440 (3.3)	17 300 (5.0)		195 340 (4.8)	15 320 (4.8)
Paternal place of birth					
Africa	35 000 (0.8)	2950 (0.9)	0.13	23 600 (0.6)	1850 (0.6)
Asia	211 580 (4.5)	18 300 (5.3)		202 960 (5.0)	15 920 (5.0)
Canada	3 698 460 (79.4)	258 740 (75.5)		3 134 880 (77.7)	245 830 (77.7)
Central and South America	57 900 (1.2)	5390 (1.6)		50 330 (1.2)	3950 (1.2)
Europe	273 180 (5.9)	18 620 (5.4)		196 280 (4.9)	15 390 (4.9)
North America excluding Canada	69 000 (1.5)	5310 (1.5)		46 880 (1.2)	3680 (1.2)
Other^b^	310 880 (6.7)	33 280 (9.7)		378 940 (9.4)	29 720 (9.4)
Maternal marital status at birth					
Married	3 476 020 (74.7)	232 010 (67.7)	0.16	2 777 070 (68.8)	217 770 (68.8)
Other	80 300 (1.7)	7510 (2.2)		65 610 (1.6)	5150 (1.6)
Missing	116 470 (2.5)	14 120 (4.1)		151 460 (3.8)	11 880 (3.8)
Single	983 200 (21.1)	88 950 (26.0)		1 039 740 (25.8)	81 540 (25.8)
Province of birth					
Alberta	527 340 (11.3)	39 250 (11.5)	0.05	463 670 (11.5)	36 360 (11.5)
Atlantic provinces	320 610 (6.9)	23 330 (6.8)		272 260 (6.7)	21 350 (6.7)
British Columbia	548 130 (11.8)	37 730 (11.0)		424 630 (10.5)	33 300 (10.5)
Manitoba	207 000 (4.4)	17 200 (5.0)		189 000 (4.7)	14 820 (4.7)
Ontario	1 740 060 (37.4)	133 040 (38.8)		1 586 220 (39.3)	124 390 (39.3)
Quebec	1 095 210 (23.5)	77 180 (22.5)		931 680 (23.1)	73 060 (23.1)
Saskatchewan	195 930 (4.2)	13 320 (3.9)		151 350 (3.8)	11 870 (3.8)
Yukon, Nunavut, and Northwest Territories	21 710 (0.5)	1530 (0.4)		15 070 (0.4)	1180 (0.4)
Birth year					
1983	317 260 (6.8)	21 250 (6.2)	0.09	256 890 (6.4)	20 150 (6.4)
1984	322 000 (6.9)	22 260 (6.5)		267 020 (6.6)	20 940 (6.6)
1985	322 210 (6.9)	21 230 (6.2)		254 650 (6.3)	19 970 (6.3)
1986	320 060 (6.9)	21 300 (6.2)		256 100 (6.3)	20 080 (6.3)
1987	319 100 (6.9)	21 720 (6.3)		259 700 (6.4)	20 370 (6.4)
1988	326 460 (7.0)	23 460 (6.8)		279 540 (6.9)	21 920 (6.9)
1989	335 800 (7.2)	23 460 (6.8)		279 970 (6.9)	21 960 (6.9)
1990	348 390 (7.5)	24 550 (7.2)		288 300 (7.1)	22 610 (7.1)
1991	349 940 (7.5)	25 600 (7.5)		298 170 (7.4)	23 380 (7.4)
1992	351 620 (7.6)	26 040 (7.6)		304 430 (7.5)	23 870 (7.5)
1993	340 040 (7.3)	26 860 (7.8)		311 560 (7.7)	24 430 (7.7)
1994	341 660 (7.3)	27 680 (8.1)		320 120 (7.9)	25 100 (7.9)
1995	336 000 (7.2)	28 680 (8.4)		330 550 (8.2)	25 920 (8.2)
1996	325 460 (7.0)	28 490 (8.3)		326 900 (8.1)	25 640 (8.1)

Abbreviation: SMD, standardized mean difference.

^a^ All numbers have been rounded to the nearest 10 to preserve confidentiality.

^b^ Includes Oceania or Antarctica and adjacent islands and missing or unknown data.

In the unmatched cohort, we estimated the incidence rate of all-cause mortality per 10 000 person-years by PTB (and GA categories) and plotted the unadjusted cumulative incidence of all-cause mortality between the ages of 1 year and 36 years by GA categories. In the matched cohorts, we compared and plotted the cumulative incidence of all-cause mortality during the first year (aged 0-11 months) and between the ages of 1 year and 36 years by PTB status. We used log-binomial regression models to calculate RRs and risk differences (RDs) for mortality between the ages of 1 year and 36 years and in narrower age ranges (ages 0-11 months and ages 1-5, 6-12, 13-17, 18-28, and 29-36 years) among individuals who were still alive at the beginning of the respective age range. These age intervals were chosen to examine associations with mortality in different stages from birth to adulthood. We also estimated and plotted RDs and RRs stratified by age in years (ages 1-36 years). We estimated all associations for PTB as a binary variable and by GA categories. For cause-specific mortality, we estimated hazard ratios (HRs) using Cox proportional hazards regression models in 4 age ranges (0-11 months and 1-5, 6-17, and 18-36 years) to have a sufficient number of events and censored individuals who died from other causes.^29^ We accounted for clustering of siblings born to the same mother using clustered variance estimates in all models. We used R, version 4.2.1 (R Project for Statistical Computing) for descriptive analyses and matching and Stata, version 16.1 (StataCorp LLC) for graphs and log-binomial regression and Cox proportional hazards regression models. Precision around point estimates was provided using 2-sided 95% CIs, and estimates were considered statistically significant if the 95% CIs did not include the null value. The threshold for statistically significance was 2-tailed *P* < .05.

Since individuals had an unequal follow-up time, we estimated HRs for all-cause mortality using Cox proportional hazards regression models in secondary analyses, with age in years (months for mortality between birth and age 11 months) as the underlying time scale and time 0 defined as the beginning of the time interval. In additional analyses, we tested for effect modification by sex and birth year and estimated associations stratified by sex and birth year for all-cause mortality. In a subsample with linkage to maternal tax records at baseline (births during 1990-1996), we additionally matched on family income quintiles (based on the average maternal family income during the 2 years before birth) and place of residence (rural or urban) and compared results.^30^

## Results

### Cohort Description

**Figure 1.**
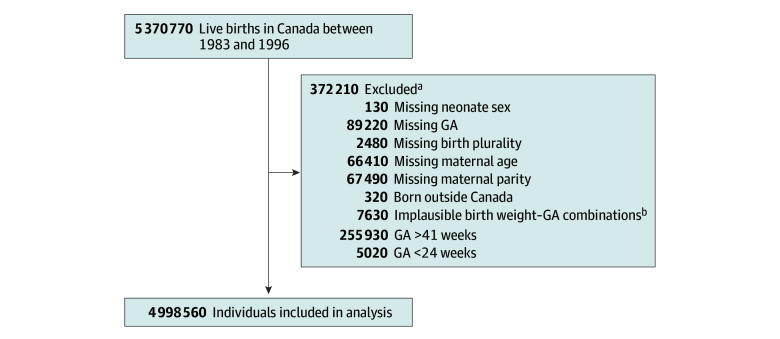
Flowchart of the Formation of the Study Cohort All numbers have been rounded to the nearest 10 to preserve confidentiality. GA indicates gestational age. ^a^There is overlap between different missing categories. ^b^Refers to birth weight for GA *z* scores outside of the 4 or more SD range.

We identified 5 370 770 live births in Canada between 1983 and 1996. After excluding 2% of births with missing information on baseline covariates or missing or invalid GA, postterm births (>41 weeks’ gestation), and births less than 24 weeks’ gestation, our cohort included 4 998 560 births (2 277 730 female [45.8%] and 2 378 250 [54.2%] male) (Figure 1). Of those, 342 580 (6.9%) were born preterm (14 130 [0.3%] at 24-27 weeks’ gestation, 30 640 [0.6%] at 28-31 weeks’ gestation, 40 560 [0.8%] at 32-33 weeks’ gestation, and 257 250 [5.1%] at 34-36 weeks’ gestation) (eTable 2 in Supplement 1). Compared with individuals who were born at term, those born preterm were more likely to be male, a twin, or a higher-order multiple birth. They were also more likely to be born to mothers younger than 20 years or 35 years or older, mothers with single or missing marital status, multiparous mothers (≥4 previous live births), fathers 40 years or older or missing data, and non–Canadian-born parents (excluding European-born parents and North America excluding Canada-born fathers) (Table 1). The matched cohort included 4 350 210 births (316 330 born preterm [7.3%] and 4 033 880 at term [92.7%]). As expected, differences in baseline characteristics were no longer evident after matching (Table 1).

### Outcome Descriptive Statistics in the Unmatched Cohorts

The median follow-up was 29 years (IQR, 26-33 years), during which 72 662 individuals (14 312 born preterm [19.7%] and 58 350 at term [80.3%]) died (eTable 3 in Supplement 1). Between 1 year and 36 years of age, the unadjusted average annual incidence of all-cause mortality was 5.94 (95% CI, 5.79-6.10) per 10 000 person-years for those born preterm and 3.73 (95% CI, 3.70-3.76) for those born at term. By age 36 years, 1.7% of individuals who were born preterm and survived their first year died (4.7% for 24-27 weeks’ GA, 2.6% for 28-31 weeks’ GA, 1.8% for 32-33 weeks’ GA, and 1.4% for 34-36 weeks’ GA categories) compared with 1.1% among those born at term. eFigure 1 in Supplement 1 depicts the crude cumulative incidence of all-cause mortality beyond infancy by GA category in the unmatched cohort and highlights that total mortality was higher with decreasing GA.

During the first year of life, the unadjusted average monthly incidence of all-cause mortality was 23.9 (95% CI, 23.4-24.42) for those born preterm and 1.82 (95% CI, 1.79-1.86) for those at term per 10 000 child-months. By the end of the first year of life, 2.6% of those born preterm died (26.2% for 24-27 weeks’ GA, 6.0% for 28-31 weeks’ GA, 2.4% for 32-33 weeks’ GA, and 0.9% for 34-36 weeks’ GA) compared with 0.2% for those born at term.

### PTB and All-Cause Mortality in the Matched Cohorts

**Figure 2.**
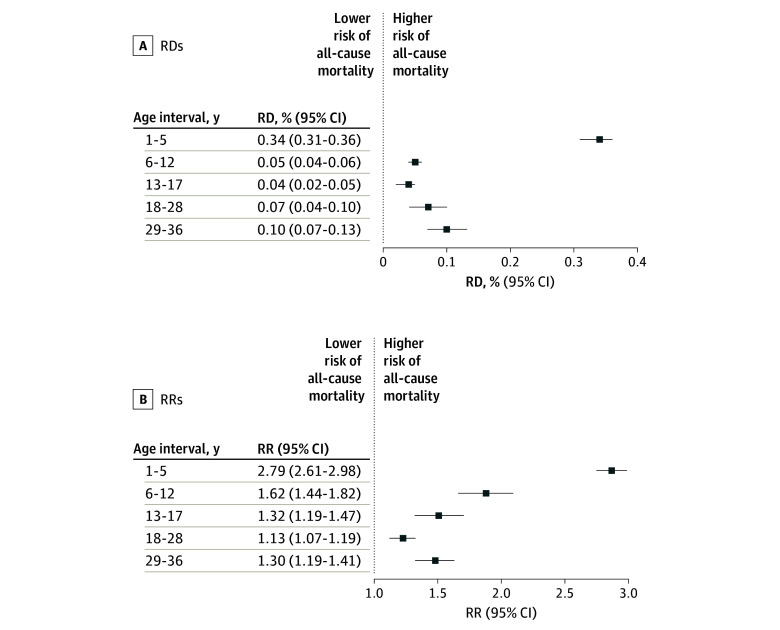
Risk Differences (RDs) and Risk Ratios (RRs) for the Association Between Preterm Birth and All-Cause Mortality in the Matched Cohort, Stratified by Age Intervals

The Kaplan-Meier plot of cumulative incidence of all-cause mortality past the age of 1 year and until age 36 years by PTB status in the matched cohort (eFigure 2 in Supplement 1) demonstrated higher mortality among individuals who were born preterm. The RD and RR for all-cause mortality between the ages of 1 year and 36 years showed positive associations between PTB and all-cause mortality in the matched cohorts (RD, 0.54% [95% CI, 0.49%-0.59%]; RR, 1.49 [95% CI, 1.44-1.54]). In analyses of narrower age intervals (Figure 2), PTB was associated with an increased risk of mortality for all age periods; the largest effect sizes were seen in early childhood (ages 1-5 years) (RD, 0.34% [95% CI, 0.31%-0.36%]; RR, 2.79 [95% CI, 2.61-2.98]) and the smallest were observed in the 18 to 28 years’ age period (RD, 0.07% [95% CI, 0.04%-0.10%]; RR, 1.13 [95% CI, 1.07-1.19]). The results by GA categories showed larger effect sizes with lower GAs, although some of the estimates were imprecise due to a small number of individuals (Table 2). eFigure 3 in Supplement 1 illustrates RDs and RRs for the association between PTB and all-cause mortality in the matched cohort, stratified by age in years (ages 1-36 years). eFigure 3 in Supplement 1 revealed that PTB was consistently associated with increased risk of mortality, with the highest risk in early years and slowly declining with age until early adulthood and then slightly increasing beyond the age of 30 years.

**Table 2.** RDs and RRs for the Association Between GA Categories and All-Cause Mortality in the Matched Cohort

Category	RD, % (95% CI)	RR (95% CI)
**Age 1-36 y**		
GA category, wk		
34-36	0.31 (0.26 to 0.36)	1.29 (1.24 to 1.34)
32-33	0.72 (0.58 to 0.87)	1.63 (1.50 to 1.78)
28-31	1.35 (1.15 to 1.54)	2.14 (1.96 to 2.33)
24-27	3.40 (2.97 to 3.83)	3.83 (3.44 to 4.27)
**Age 0-11 mo**		
GA category, wk		
34-36	0.67 (0.64 to 0.71)	4.22 (3.98 to 4.48)
32-33	2.25 (2.09 to 2.41)	10.70 (9.60 to 11.92)
28-31	5.76 (5.47 to 6.04)	24.91 (22.41 to 27.69)
24-27	25.43 (24.65 to 26.21)	105.79 (87.61 to 127.73)
**Age 1-5 y**		
GA category, wk		
34-36	0.15 (0.12 to 0.17)	1.81 (1.67 to 1.96)
32-33	0.49 (0.40 to 0.58)	3.41 (2.87 to 4.04)
28-31	0.97 (0.84 to 1.10)	6.00 (5.20 to 6.93)
24-27	2.70 (2.36 to 3.04)	15.73 (13.33 to 18.55)
**Age 6-12 y**		
GA category, wk		
34-36	0.03 (0.02 to 0.05)	1.42 (1.24 to 1.63)
32-33	0.08 (0.03 to 0.12)	1.95 (1.47 to 2.60)
28-31	0.11 (0.05 to 0.17)	2.26 (1.60 to 3.19)
24-27	0.20 (0.09 to 0.31)	3.38 (2.17 to 5.27)
**Age 13-17 y**		
GA category, wk		
34-36	0.02 (0.01 to 0.04)	1.21 (1.07 to 1.36)
32-33	0.02 (−0.03 to 0.06)	1.14 (0.84 to 1.55)
28-31	0.13 (0.06 to 0.19)	1.96 (1.44 to 2.66)
24-27	0.23 (0.11 to 0.35)	3.06 (2.09 to 4.46)
**Age 18-28 y**		
GA category, wk		
34-36	0.06 (0.02 to 0.09)	1.11 (1.04 to 1.17)
32-33	0.09 (0 to 0.17)	1.15 (1.00 to 1.32)
28-31	0.11 (0.01 to 0.22)	1.20 (1.02 to 1.40)
24-27	0.31 (0.11 to 0.51)	1.51 (1.20 to 1.89)
**Age 29-36 y**		
GA category, wk		
34-36	0.10 (0.06 to 0.14)	1.30 (1.18 to 1.43)
32-33	0.12 (0.02 to 0.22)	1.36 (1.09 to 1.70)
28-31	0.09 (−0.02 to 0.21)	1.26 (0.97 to 1.63)
24-27	0.05 (−0.15 to 0.24)	1.12 (0.71 to 1.76)

Abbreviations: GA, gestational age; RD, risk difference; RR, risk ratio.

### PTB and Cause-Specific Mortality in the Matched Cohorts

For cause-specific mortality, PTB was associated with an increased risk of mortality (ages 1-36 years) associated with respiratory, circulatory, and digestive system disorders; nervous system, endocrine, and infectious diseases; cancers; congenital malformations; and conditions originating in the perinatal period (Table 3). The highest HRs were observed for conditions originating in the perinatal period (HR, 37.50 [95% CI, 29.80-47.20]) and for digestive and respiratory disorders, nervous system diseases, congenital malformations, and infection (HRs ranged from 2.34 [95% CI, 1.94-2.83] to 3.27 [95% CI, 2.97-3.61]). No associations for external causes of mortality were observed. These patterns were generally consistent across age groups (ages 1-5, 6-17, and 18-36 years) and GA categories, with higher HRs among lower age groups and lower GAs (eTable 4 in Supplement 1).

**Table 3.** Associations Between Preterm Birth and Cause-Specific Mortality Between Ages 1 Year and 36 Years in the Matched Cohort

Outcome	Term birth (n = 4 026 050)		Preterm birth (n = 308 400)		HR (95% CI)
	No. of deaths^a^	Absolute risk per 10 000 persons	No. of deaths^a^	Absolute risk per 10 000 persons	
Respiratory system disorders	970	2.4	230	7.5	2.95 (2.56-3.41)
Circulatory disorders	1650	4.3	230	7.5	1.81 (1.58-2.08)
Diseases of the nervous system	1730	4.1	410	13.3	2.90 (2.61-3.23)
Infectious diseases	560	1.4	120	3.9	2.63 (2.16-3.20)
Digestive system disorders	680	1.7	130	4.2	2.34 (1.94-2.83)
Endocrine, nutritional, and metabolic diseases	1000	2.5	120	3.9	1.60 (1.32-1.94)
Cancer	3980	9.9	340	11.0	1.19 (1.07-1.33)
External causes of mortality	24 910	61.9	2110	68.4	1.02 (0.98-1.07)
Mental and behavioral disorders	370	0.9	40	1.3	1.32 (0.95-1.84)
Congenital malformation	1910	4.7	510	16.5	3.27 (2.97-3.61)
Conditions originating in the perinatal period	90	0.2	280	9.1	37.50 (29.80-47.20)

Abbreviation: HR, hazard ratio.

^a^ The number of deaths has been rounded to the nearest 10 to preserve confidentiality.

### PTB and Mortality Among Newborns and Infants in the Matched Cohorts

We found an increased risk of all-cause mortality associated with PTB among newborns and infants (ages 0 to 11 months) (RD, 2.29 [95% CI, 2.23-2.35]; RR, 11.61 [95% CI, 11.09-12.15]), with higher RDs and RRs among those with lower GAs (Table 2 and eFigure 4 in Supplement 1). For cause-specific mortality, higher HRs were observed for mortality associated with conditions originating in the perinatal period (HR, 43.20 [95% CI, 40.57-46.00]) followed by digestive system disorders, congenital malformations, and infections (HRs ranged from 8.46 [95% CI, 6.3-11.36] to 13.38 [95% CI, 9.58-18.69]) (eTable 5 in Supplement 1).

### Secondary Analyses

HRs for associations between PTB and all-cause mortality were nearly identical to RRs across different age periods (eTable 6 in Supplement 1). In sex-stratified analyses, RDs were slightly higher among males, and RRs were generally lower among males across age groups (eFigure 5 and eTable 7 in Supplement 1). No evidence of effect modification by birth year was found for the associations between PTB and all-cause mortality between ages 1 year and 36 years (eTable 8 in Supplement 1). For all-cause mortality in the first year, results suggested lower RDs in more recent birth years. Associations between PTB and all-cause mortality, after accounting for family income and rural residence at baseline, along with other baseline characteristics, were similar to the main results (eTable 9 in Supplement 1).

## Discussion

In this large population-based cohort study, individuals born preterm were at increased risk of mortality from birth to the ages of 23 to 36 years. The risk of death was the highest in early years and declined with age, rising slightly beyond the age of 30 years. We identified higher risk of death as GA at birth decreased, with those born less than 28 weeks’ gestation having the highest risk of mortality across all age intervals. Additionally, PTB was found to be associated with an increased risk of mortality related to respiratory, circulatory, and digestive system disorders; nervous system, endocrine, and infectious diseases; cancers; congenital malformations; and perinatal conditions.

PTB can disrupt the normal progression of intrauterine growth and maturation in all fetal organs, predisposing individuals who are affected to a range of morbidities and chronic diseases.^11^ For example, neonates who are born preterm are at increased risk of bronchopulmonary dysplasia, which is associated with subsequent chronic lung diseases.^31,32,33^ Necrotizing enterocolitis is also common among neonates born preterm, particularly those born at less than 28 weeks’ gestation, and it predisposes these children to a range of gastrointestinal problems later in life.^34,35^ PTB also can lead to a range of neurological impairments, including cerebral palsy and epilepsy.^36,37^ PTB is also associated with adiposity in early childhood and adolescence, which can put these individuals at risk of cardiometabolic complications.^38,39,40^ PTB has also been associated with select pediatric cancers, such as hepatoblastoma and nephroblastoma.^41,42^ Some underlying conditions that predispose to PTB may also increase the risk of mortality, such as congenital malformations.^43,44^ Furthermore, a growing literature has shown an increased risk of several chronic illnesses, including diabetes, ischemic health diseases, heart failure, hypertension, asthma, stroke, mental and behavioral problems, and infections later in life among individuals who are born preterm.^5,6,45,46,47,48,49,50,51,52,53,54,55^ Findings for cause-specific mortality are also aligned with these biologic effects of PTB on various body systems across the lifespan.^5,6^

In a systematic review by Crump et al^10^ that included 8 studies (6 Swedish, 1 Australian, and 1 Norwegian), RRs for the association between PTB and adulthood mortality ranged between 1.2 and 1.6 (1.9-4.0 for extremely PTB groups). These estimates were adjusted for various sociodemographic and health factors, with some studies using a co-sibling design. Our estimates were lower than those reported in the review, primarily for newborns and infants in the less than 28 weeks’ gestation group, likely due to variation in maximum age attained (ranged between 30 and 95 years between studies), latest follow-up year (2000-2017), and the definition of the extremely PTB group (24-27 weeks’ gestation in our study vs 22-27 weeks’ gestation in the review). We chose to restrict to 24 weeks’ gestation or above because of the known issue of underreporting of perinatal deaths for extremely PTB groups in Canada.^21,22^

Consistent with our results, a recent study conducted in 4 Nordic countries^13^ examined the risk of mortality between the ages of 15 and 51 years and found increased risk of mortality for those born less than 34 weeks’ gestation (adjusted HR, 1.44 [95% CI, 1.34-1.55]; adjusted for birth year, sex, birth weight, congenital malformations, maternal age, and parity) and for those born at 34 to 36 weeks’ gestation (adjusted HR, 1.23 [95% CI, 1.18-1.29]). Similarly, the study found increased risk of mortality related to cardiovascular diseases, diabetes, and respiratory diseases.^13^ Our findings are also consistent with earlier studies focusing on the association between birth weight and mortality in adulthood, which found higher risk of mortality among children with low birth-weight (<2.5 kg).^10,56^ However, while the majority of the low birth-weight category includes PTBs, low birth weight is a composite metric influenced by both GA and fetal growth. As a result, the at-risk category of low birth weight encompasses both preterm neonates and neonates who were small for GA born at later gestations. ^10,57^

Analyses stratified by birth year revealed a slightly decreased risk of mortality during the first year of life among more recent cohorts (those born after 1990 compared with those born in the early 1980s). This is consistent with a Swedish study that found improved infant mortality rates after PTB between 1973 and 2008,^58^ as well as a Norwegian study that documented decreased neonatal mortality among neonates who were born preterm between 1967 and 1983.^59^ These findings may reflect improvements in survival due to changes in neonatal care over time.

Sex-stratified analyses revealed slightly higher RDs but lower RRs among males across age groups, indicating a positive additive interaction and a negative multiplicative interaction between PTB and sex. This is likely attributed to the higher baseline risk of mortality in males, irrespective of GA. Existing literature supports male sex as a risk factor for various complications associated with PTB, including congenital malformations, respiratory complications, and neurodevelopmental deficits.^60,61,62^ Furthermore, injury-associated mortality risk has been shown to be higher in adolescent and young males than in females.^63,64^ Similar to our findings, studies have reported higher RDs among males.^11,13,15^ The Risnes et al^13^ study also found lower RRs for males, while other studies found similar RRs between males and females.^9,12^

### Implications and Directions for Future Research

These findings contribute to the growing evidence of the diverse adverse effects of PTB throughout a lifespan. Aligned with conclusions from previous reviews and international expert panels,^65,66^ these results suggest the need for long-term follow-up for individuals who are born preterm for proactive and therapeutic measures. Regular inquiries about birth history during medical consultations, counseling for patients, screening for chronic diseases in those born preterm, and promptly addressing emerging complications may be beneficial to reduce the risk of mortality. There is a need for future studies that find effective interventions and protective factors that protect children born preterm from premature mortality. Future studies should consider longer follow-up time into late adulthood, when risks for chronic diseases and mortality increase. Additionally, examining mortality among birth cohorts born after recent advances in perinatal care can help determine if the long-term mortality risk following PTB has diminished. Studies in other populations are also essential, particularly in low-income and middle-income countries, in which a substantial burden of PTBs occurs. In addition, there is a need for studies that examine potential mediators (eg, neonatal morbidities) for these observed associations.

### Strengths and Limitations

Among this study’s strengths is the population-level data linkage that allowed for long follow-up from birth to 36 years of age. Additionally, study cohort and outcome data were obtained from vital statistics registries with high levels of completeness.^67^

This study also has several limitations. Birth records relied on physician or parent-reported GA data and thus were vulnerable to measurement errors.^20^ However, the Canadian Vital Statistics—Birth database undergoes rigorous error detection and data validation procedures, which include both provincial edits and extensive checks by Statistics Canada to enhance the quality and accuracy of the data. Statistical power was limited for some analyses stratified by GA and for cause-specific analyses, particularly in the oldest age group. The study included births that occurred in the 1980s through the 1990s and therefore may not be generalizable to recent births, in which advances in perinatal care have substantially improved survival. However, our analyses separately considered mortality after 1 year of age, and thus improved survival in a neonatal unit would not have affected these results. Additionally, including historical birth records enabled examining associations with mortality up to the fourth decade of life. Although we used matching to control for differences in several demographic factors, we cannot rule out residual confounding by unmeasured confounders. For example, we did not have information on maternal health and comorbidities, the type of PTB (spontaneous vs iatrogenic), the presence of congenital malformations, and maternal socioeconomic and lifestyle factors. We matched on marital status as a proxy for social support and socioeconomic status. However, the significance of marital status may have evolved over time due to societal changes, but data available on birth certificates on maternal marital status were limited, as they only considered legal marital status and did not differentiate cohabiting or common-law partner families. Our data may have missed some deaths occurring in the early neonatal period, particularly those born near the threshold of viability.^21,22^

## Conclusions

The findings of this cohort study suggest that individuals born preterm were at increased risk of death from birth to their third and fourth decades of life, and the risks were higher with decreasing GA at birth. Some of these associations may have been partly due to underlying health determinants that affected both PTB and mortality. These findings suggest that PTB should be recognized as a risk factor for mortality and could inform preventive strategies. Additionally, they highlight the need for further follow-up studies to assess possible adverse consequences of PTB into adulthood.

## Notes

Supplement 1. **eTable 1.** List of *International Classification of Diseases, Ninth Revision* (*ICD-9*) and *International Statistical Classification of Diseases and Related Health Problems, Tenth Revision* (*ICD-10*) Codes Used to Classify Cause-Specific Mortality **eTable 2.** Characteristics of the Overall Cohort (N = 4 998 560), N (%) **eTable 3** Descriptive Statistics for All-Cause Mortality by Preterm Birth (and Gestational Age Categories) in the Unmatched Cohort **eTable 4.** Hazard Ratios With 95% CIs for the Association Between Preterm Birth and Selected Cause-Specific Mortality in the Matched Cohort, Stratified by Age Intervals **eTable 5.** Associations Between Preterm Birth and Cause-Specific Mortality Between Birth and 11 Months in the Matched Cohort **eTable 6.** Hazard Ratios With 95% CIs for the Association Between PTB and All-Cause Mortality in the Matched Cohort **eTable 7.** Risk Differences (RDs, %) and Ratios (RRs) With 95% CIs for the Association Between PTB and All-Cause Mortality From Birth to 11 Months of Age in the Matched Cohort, Stratified by Sex **eTable 8.** Risk Differences (RDs, %) and Ratios (RRs) With 95% CIs for the Association Between PTB and All-Cause Mortality in the Matched Cohort, Stratified by Birth Year **eTable 9.** Risk Differences (RDs, %) and Ratios (RRs) With 95% CIs for the Association Between PTB and All-Cause Mortality in the Matched Cohort Among a Subsample Linked to Maternal Tax With Matching on Family Income and Rural Residence (1990-1996 Births) **eFigure 1**. Kaplan-Meier Plot of Cumulative Incidence of All-Cause Mortality by Gestational Age Categories in the Unmatched Cohort (Ages 1-36 Years) **eFigure 2.** Kaplan-Meier Plot of Cumulative Incidence of All-Cause Mortality by Preterm Birth in the Matched Cohort (Ages 1-36 Years) **eFigure 3.** Risk Differences (RDs, %) and Ratios (RRs) for the Association Between Preterm Birth and All-Cause Mortality in the Matched Cohort, Stratified by Age in Years (Ages 1-36 Years) **eFigure 4.** Kaplan-Meier Plot of Cumulative Incidence of All-Cause Mortality by Preterm Birth in the Matched Cohort (Ages 0-11 Months) **eFigure 5.** Risk Differences (RDs, %) and Ratios (RRs) With 95% CIs for the Association Between PTB and All-Cause Mortality in the Matched Cohort, Stratified by Sex

Supplement 2.
Data Sharing Statement
