# Supplementary material for: Short-Term and Long-Term Mortality Risk After Preterm Birth
Source: JAMA Netw Open. 2024 Nov 20;7(11):e2445871. doi: 10.1001/jamanetworkopen.2024.45871 (PMC11579792; doi:10.1001/jamanetworkopen.2024.45871)
Supplement: Supplement 1. — eTable 1. List of International Classification of Diseases, Ninth Revision (ICD-9) and International Statistical Classification of Diseases and Related Health Problems, Tenth Revision (ICD-10) Codes Used to Classify Cause-Specific Mortality eTable 2. Characteristics of the Overall Cohort (N = 4 998 560), N (%) eTable 3 Descriptive Statistics for All-Cause Mortality by Preterm Birth (and Gestational Age Categories) in the Unmatched Cohort eTable 4. Hazard Ratios With 95% CIs for the Association Between Preterm Birth and Selected Cause-Specific Mortality in the Matched Cohort, Stratified by Age Intervals eTable 5. Associations Between Preterm Birth and Cause-Specific Mortality Between Birth and 11 Months in the Matched Cohort eTable 6. Hazard Ratios With 95% CIs for the Association Between PTB and All-Cause Mortality in the Matched Cohort eTable 7. Risk Differences (RDs, %) and Ratios (RRs) With 95% CIs for the Association Between PTB and All-Cause Mortality From Birth to 11 Months of Age in the Matched Cohort, Stratified by Sex eTable 8. Risk Differences (RDs, %) and Ratios (RRs) With 95% CIs for the Association Between PTB and All-Cause Mortality in the Matched Cohort, Stratified by Birth Year eTable 9. Risk Differences (RDs, %) and Ratios (RRs) With 95% CIs for the Association Between PTB and All-Cause Mortality in the Matched Cohort Among a Subsample Linked to Maternal Tax With Matching on Family Income and Rural Residence (1990-1996 Births) eFigure 1. Kaplan-Meier Plot of Cumulative Incidence of All-Cause Mortality by Gestational Age Categories in the Unmatched Cohort (Ages 1-36 Years) eFigure 2. Kaplan-Meier Plot of Cumulative Incidence of All-Cause Mortality by Preterm Birth in the Matched Cohort (Ages 1-36 Years) eFigure 3. Risk Differences (RDs, %) and Ratios (RRs) for the Association Between Preterm Birth and All-Cause Mortality in the Matched Cohort, Stratified by Age in Years (Ages 1-36 Years) eFigure 4. Kaplan-Meier Plot of Cumulative Incidence of All-Cau [file jamanetwopen-e2445871-s001.pdf]

Supplemental Online Content

Ahmed AM, Grandi SM, Pullenayegum E, et al. Short-term and long-term mortality risk after preterm birth. *JAMA Netw Open*. 2024;7(11):e2445871. doi:10.1001/jamanetworkopen.2024.45871

eTable 1. List of *International Classification of Diseases, Ninth Revision* (*ICD-9*) and *International Statistical Classification of Diseases and Related Health Problems, Tenth Revision* (*ICD-10*) Codes Used to Classify Cause-Specific Mortality
eTable 2. Characteristics of the Overall Cohort (N = 4 998 560), N (%)
eTable 3 Descriptive Statistics for All-Cause Mortality by Preterm Birth (and Gestational Age Categories) in the Unmatched Cohort
eTable 4. Hazard Ratios With 95% CIs for the Association Between Preterm Birth and Selected Cause-Specific Mortality in the Matched Cohort, Stratified by Age Intervals
eTable 5. Associations Between Preterm Birth and Cause-Specific Mortality Between Birth and 11 Months in the Matched Cohort
eTable 6. Hazard Ratios With 95% CIs for the Association Between PTB and All-Cause Mortality in the Matched Cohort
eTable 7. Risk Differences (RDs, %) and Ratios (RRs) With 95% CIs for the Association Between PTB and All-Cause Mortality From Birth to 11 Months of Age in the Matched Cohort, Stratified by Sex
eTable 8. Risk Differences (RDs, %) and Ratios (RRs) With 95% CIs for the Association Between PTB and All-Cause Mortality in the Matched Cohort, Stratified by Birth Year
eTable 9. Risk Differences (RDs, %) and Ratios (RRs) With 95% CIs for the Association Between PTB and All-Cause Mortality in the Matched Cohort Among a Subsample Linked to Maternal Tax With Matching on Family Income and Rural Residence (1990-1996 Births)
eFigure 1. Kaplan-Meier Plot of Cumulative Incidence of All-Cause Mortality by Gestational Age Categories in the Unmatched Cohort (Ages 1-36 Years)
eFigure 2. Kaplan-Meier Plot of Cumulative Incidence of All-Cause Mortality by Preterm Birth in the Matched Cohort (Ages 1-36 Years)
eFigure 3. Risk Differences (RDs, %) and Ratios (RRs) for the Association Between Preterm Birth and All-Cause Mortality in the Matched Cohort, Stratified by Age in Years (Ages 1-36 Years)
eFigure 4. Kaplan-Meier Plot of Cumulative Incidence of All-Cause Mortality by Preterm Birth in the Matched Cohort (Ages 0-11 Months)
eFigure 5. Risk Differences (RDs, %) and Ratios (RRs) With 95% CIs for the Association Between PTB and All-Cause Mortality in the Matched Cohort, Stratified by Sex

This supplemental material has been provided by the authors to give readers additional information about their work.

© 2024 Ahmed AM et al. *JAMA Network Open*.

eTable 1. List of *International Classification of Diseases, Ninth Revision* (*ICD-9*) and *International Statistical Classification of Diseases and Related Health Problems, Tenth Revision* (*ICD-10*) Codes Used to Classify Cause-Specific Mortality

**Cause of death**	***ICD-9***	***ICD-10***
Respiratory system disorders	460-519	J00-J98
Circulatory disorders	390-459	I00-I99
Diseases of the nervous system	320-359	G06-G98
Infectious diseases	001-139	A00-B99
Digestive system disorders	520-579	K00-K92
Endocrine, nutritional and metabolic diseases	240-279	E03-E88
Cancer	140-239	C00-D48
External causes of mortality	800-999	V00-Y89
Mental and Behavioral disorders	290-319	F00-F99
Congenital malformation	740-759	Q00-Q99
Conditions originating in the perinatal period	760-779	P00-P96

© 2024 Ahmed AM et al. *JAMA Network Open*.

eTable 2. Characteristics of the Overall Cohort (N = 4 998 560), N (%)

Characteristics	N (%)
Individual's sex	
Female	2434500 (48.7)
Male	2564060 (51.3)
Birth plurality	
Singleton	4890470 (97.8)
Multiple	108090 (2.2)
Maternal parity	
≥4	108490 (2.2)
0	2157990 (43.2)
1	1778300 (35.6)
2	733620 (14.7)
3	220170 (4.4)
Maternal age	
<20 years	303710 (6.1)
≥40 years	52540 (1.1)
20-24 years	1107710 (22.2)
25-29 years	1852140 (37.1)
30-34 years	1284080 (25.7)
35-39 years	398380 (8)
Paternal age	
<25 years	598220 (12)
≥40 years	290540 (5.8)
25–29 years	1512720 (30.3)
30–34 years	1519820 (30.4)
35–39 years	700300 (14)
Missing	376950 (7.5)
Maternal place of birth	
Africa	33000 (0.7)
Asia	233700 (4.7)
Canada	4156270 (83.1)
Central and South America	64850 (1.3)
Europe	250470 (5)
North America	91540 (1.8)
Other	168730 (3.4)
Paternal place of birth	
Africa	37950 (0.8)
Asia	229870 (4.6)
Canada	3957190 (79.2)
Central and South America	63290 (1.3)
Europe	291800 (5.8)
North America	74300 (1.5)
Other	344160 (6.9)
Maternal marital status at birth	
Married	3708030 (74.2)
Other	130590 (2.6)
Missing	87810 (1.8)
Single	1072140 (21.4)
Place of birth	
Alberta	566590 (11.3)
Atlantic Provinces	343940 (6.9)
British Columbia	585860 (11.7)
Manitoba	224190 (4.5)
Ontario	1873090 (37.5)
Quebec	1172380 (23.5)
Saskatchewan	209260 (4.2)
Territories	23240 (0.5)
Birth year	

© 2024 Ahmed AM et al. *JAMA Network Open*.

1983	338510 (6.8)
1984	344260 (6.9)
1985	343440 (6.9)
1986	341350 (6.8)
1987	340820 (6.8)
1988	349910 (7)
1989	359270 (7.2)
1990	372950 (7.5)
1991	375540 (7.5)
1992	377660 (7.6)
1993	366910 (7.3)
1994	369340 (7.4)
1995	364670 (7.3)
1996	353940 (7.1)
Preterm birth	
Preterm 24-36	342580 (6.9)
Term 37-41	4655980 (93.1)
Gestational age categories	
37-41	4655980 (93.1)
34-36	257250 (5.1)
32-33	40560 (0.8)
28-31	30640 (0.6)
24-27	14130 (0.3)

Note: All numbers were rounded to the nearest ten for confidentiality reasons

© 2024 Ahmed AM et al. *JAMA Network Open*.

eTable 3. Descriptive Statistics for All-Cause Mortality by Preterm Birth (and Gestational Age Categories) in the Unmatched Cohort

a) Age 0-11 months

Category	deaths	Person-month	Incidence rate per 10,000 person-month (95% CI)	% died by the end of first year of life
Preterm birth				
Preterm (24-36 weeks)	8,795	3,677,535	23.9 (23. 4,24.42)	2.57
Term (37-41 weeks)	9,316	51,129,814	1.82 (1.79, 1.86)	0.20
Gestational age category				
34-36 weeks	2,266	2,807,278	8.07 (7.75, 8.41)	0.88
32-33 weeks	991	436,125	22.72 (21.35, 24.18)	2.44
28-31 weeks	1,841	317,924	57.91 (55.32, 60.61)	6.01
24-27 weeks	3,697	116,208	318.14 (308.04, 328.56)	26.17

b) Age 1-36 years

Category	deaths	Person-years	Incidence rate per 10,000 person-year (95% CI)	% died by the end of follow-up
Preterm birth				
Preterm (24-36 weeks)	5,517	9,284,798	5.94 (5.79, 6.10)	1.65
Term (37-41 weeks)	49,034	131,500,000	3.73 (3.70, 3.76)	1.06
Gestational age category				
34-36 weeks	3,575	7,096,468	5.04 (4.88, 5.21)	1.40
32-33 weeks	722	1,104,420	6.54 (6.08, 7.03)	1.82
28-31 weeks	735	800,065	9.19 (8.55, 9.88)	2.55
24-27 weeks	485	283,845	17.09 (15.63, 18.68)	4.65

© 2024 Ahmed AM et al. *JAMA Network Open*.

eTable 4. Hazard Ratios With 95% CIs for the Association Between Preterm Birth and Selected Cause-Specific Mortality in the Matched Cohort, Stratified by Age Intervals

	**Respiratory system disorder**		
	**1-5 years**	**6-17 years**	**18-36 years**
Preterm birth<br>Preterm (<37 weeks)	3.67 (3.00, 4.48)	2.56 (1.83, 3.59)	2.20 (1.69, 2.87)
Gestational age category			
34-36 weeks	2.04 (1.52, 2.73)	2.17 (1.44, 3.26)	1.72 (1.24, 2.39)
32-33 weeks	5.54 (3.70, 8.31)	2.68 (1.10, 6.54)	2.81 (1.45, 5.47)
28-31 weeks	8.2 (5.62, 11.96)	2.54 (0.93, 6.89)	3.96 (2.10, 7.44)
24-27 weeks	20.97 (13.1, 33.55)	9.2 (4.02, 21.04)	8.00 (3.54, 18.05)
	**Circulatory disorders**		
Preterm birth<br>Preterm (<37 weeks)	2.42 (1.78, 3.28)	1.66 (1.22, 2.25)	1.64 (1.37, 1.96)
Gestational age category			
34-36 weeks	1.68 (1.11, 2.53)	2.41 (1.71, 3.39)	1.65 (1.35, 2.02)
32-33 weeks	2.3 (1.14, 4.64)	1.84 (0.91, 3.72)	1.13 (0.62, 2.05)
28-31 weeks	5.75 (2.82, 11.72)	-	2.17 (1.30, 3.61)
24-27 weeks	15.39 (7.52, 31.49)	1.00 (0.14, 7.18)	1.79 (0.67, 4.8)
	**Diseases of the nervous system**		
Preterm birth<br>Preterm (<37 weeks)	2.63 (2.15, 3.22)	3.52 (2.91, 4.26)	2.55 (2.16, 3.02)
Gestational age category			
34-36 weeks	1.86 (1.43, 2.43)	2.21 (1.69, 2.88)	1.83 (1.47, 2.29)
32-33 weeks	2.33 (1.39, 3.89)	3.57 (2.09, 6.1)	2.42 (1.51, 3.86)
28-31 weeks	6.04 (3.76, 9.71)	9.66 (6.88, 13.55)	5.28 (3.66, 7.61)
24-27 weeks	18.64 (11.67, 29.76)	9.22 (5.99, 14.19)	11.48 (7.75, 17.03)
	**Infectious diseases**		
Preterm birth<br>Preterm (<37 weeks)	3.62 (2.74, 4.79)	2.70 (1.80, 4.04)	1.48 (0.99, 2.22)
Gestational age category			
34-36 weeks	2.53 (1.73, 3.69)	2.66 (1.68, 4.23)	1.14 (0.68, 1.93)
32-33 weeks	3.98 (2.11, 7.50)	2.82 (1.04, 7.64)	2.13 (0.95, 4.79)
28-31 weeks	9.25 (5.25, 16.31)	2.89 (0.91, 9.16)	2.22 (0.71, 6.97)
24-27 weeks	8.58 (4.41, 16.69)	3.79 (0.52, 27.55)	5.93 (1.46, 24.13)
	**Digestive system disorders**		
Preterm birth<br>Preterm (<37 weeks)	3.74 (2.66, 5.25)	3.30 (2.08, 5.23)	1.70 (1.30, 2.21)
Gestational age category			
34-36 weeks	1.55 (0.92, 2.64)	2.00 (1.04, 3.86)	1.53 (1.12, 2.08)
32-33 weeks	8.57 (3.93, 18.66)	2.46 (0.78, 7.77)	1.05 (0.43, 2.54)
28-31 weeks	14.22 (7.58, 26.7)	7.75 (2.77, 21.67)	2.63 (1.30, 5.31)
24-27 weeks	33.8 (17.39, 65.71)	31.95 (13.28, 76.84)	8.33 (3.69, 18.8)

© 2024 Ahmed AM et al. *JAMA Network Open*.

	Endocrine, nutritional, and metabolic diseases		
Preterm birth<br>Preterm (<37 weeks)	2.04 (1.39, 3.00)	1.05 (0.69, 1.62)	1.66 (1.28, 2.14)
Gestational age category			
34-36 weeks	1.57 (0.98, 2.51)	1.07 (0.65, 1.74)	1.65 (1.22, 2.22)
32-33 weeks	5.8 (2.83, 11.88)	0.69 (0.17, 2.79)	2.30 (1.18, 4.45)
28-31 weeks	1.12 (0.16, 8.06)	1.54 (0.49, 4.83)	1.50 (0.67, 3.36)
24-27 weeks	5.74 (1.41, 23.35)	1.67 (0.23, 11.96)	1.30 (0.42, 4.04)
	Cancer		
Preterm birth<br>Preterm (<37 weeks)	1.56 (1.2, 2.03)	0.96 (0.77, 1.18)	1.19 (1.02, 1.38)
Gestational age category			
34-36 weeks	1.33 (0.97, 1.83)	0.93 (0.73, 1.2)	1.26 (1.07, 1.48)
32-33 weeks	2.24 (1.2, 4.2)	1.46 (0.89, 2.39)	1.10 (0.71, 1.72)
28-31 weeks	2.3 (1.14, 4.64)	0.93 (0.46, 1.87)	0.76 (0.41, 1.41)
24-27 weeks	2.68 (0.86, 8.36)	-	0.96 (0.4, 2.3)
	External causes of mortality		
Preterm birth<br>Preterm (<37 weeks)	1.20 (1.04, 1.38)	0.87 (0.78, 0.97)	1.01 (0.96, 1.06)
Gestational age category			
34-36 weeks	1.19 (1.02, 1.4)	0.91 (0.8, 1.03)	1.04 (0.98, 1.1)
32-33 weeks	1.47 (1.03, 2.1)	0.96 (0.7, 1.33)	0.99 (0.85, 1.14)
28-31 weeks	0.75 (0.44, 1.3)	0.64 (0.45, 0.91)	0.92 (0.77, 1.1)
24-27 weeks	1.83 (0.98, 3.4)	0.62 (0.33, 1.14)	0.74 (0.54, 1.01)
	Congenital malformation		
Preterm birth<br>Preterm (<37 weeks)	3.63 (3.23, 4.08)	2.88 (2.26, 3.68)	2.49 (1.96, 3.18)
Gestational age category		-	-
34-36 weeks	2.78 (2.41, 3.22)		
32-33 weeks	4.64 (3.53, 6.09)		
28-31 weeks	6.87 (5.24, 9.00)		
24-27 weeks	13.34 (9.49, 18.76)		
	Conditions originating in the perinatal period		
Preterm birth<br>Preterm (<37 weeks)	53.12 (40.65, 69.42)	-	-
Gestational age category		-	-
34-36 weeks	6.71 (4.26, 10.59)		
32-33 weeks	44.24 (26.34, 74.31)		
28-31 weeks	143.23 (104, 197.27)		
24-27 weeks	1049.13 (701.23, 1569.64)		

Note: Some estimates were not reported due to small cell count.

© 2024 Ahmed AM et al. *JAMA Network Open*.

eTable 5. Associations Between Preterm Birth and Cause-Specific Mortality Between Birth and 11 Months in the Matched Cohort

	Term (n=4,033,880)		Preterm (n=316,330)		
Outcome	Number of deaths	Absolute risk per 10,000	Number of deaths	Absolute risk per 10,000	Hazard ratio
Respiratory system disorders	240	0.6	100	3.2	4.05 (3.23, 5.08)
Circulatory disorders	150	0.4	60	1.9	5.15 (3.8, 6.99)
Diseases of the nervous system	230	0.6	90	2.8	5.16 (4.04, 6.59)
Infectious diseases	110	0.3	70	2.2	8.46 (6.3, 11.36)
Digestive system disorders	80	0.2	70	2.2	13.38 (9.58, 18.69)
Endocrine, nutritional and metabolic diseases	120	0.3	70	2.2	6.20 (4.66, 8.25)
Cancer	50	0.1	20	0.6	5.61 (3.28, 9.62)
External causes of mortality	340	0.8	60	1.9	2.31 (1.77, 3.02)
Congenital malformation	3,210	0.8	2,550	80.6	9.81 (9.32, 10.33)
Conditions originating in the perinatal period	1,110	2.5	4,270	135.0	43.2 (40.57, 46.00)

© 2024 Ahmed AM et al. *JAMA Network Open*.

eTable 6. Hazard Ratios With 95% CIs for the Association Between PTB and All-Cause Mortality in the Matched Cohort

Category	**Age 1-36 years**	
Preterm birth		
Preterm (<37 weeks)	1.49 (1.45, 1.54)	
Gestational age category		
34-36 weeks	1.29 (1.24, 1.34)	
32-33 weeks	1.64 (1.52, 1.77)	
28-31 weeks	2.17 (2.01, 2.34)	
24-27 weeks	3.98 (3.62, 4.37)	
	**Age 0-11 months**	**Age 1-5 years**
Preterm birth		
Preterm (<37 weeks)	11.72 (11.36, 12.08)	2.80 (2.65, 2.95)
Gestational age category		
34-36 weeks	4.24 (4.04, 4.44)	1.81 (1.68, 1.95)
32-33 weeks	10.82 (10.10, 11.59)	3.41 (3.01, 3.88)
28-31 weeks	25.62 (24.27, 27.04)	6.03 (5.37, 6.77)
24-27 weeks	119.31 (114.16, 124.69)	15.9 (14.06, 17.99)
	**Age 6-12 years**	**Age 13-17 years**
Preterm birth		
Preterm (<37 weeks)	1.62 (1.46, 1.80)	1.32 (1.20, 1.45)
Gestational age category		
34-36 weeks	1.42 (1.26, 1.61)	1.21 (1.08, 1.35)
32-33 weeks	1.95 (1.50, 2.55)	1.14 (0.86, 1.50)
28-31 weeks	2.26 (1.71, 2.99)	1.96 (1.54, 2.50)
24-27 weeks	3.38 (2.30, 4.98)	3.06 (2.15, 4.36)
	**Age 18-28 years**	**Age 29-36 years**
Preterm birth		
Preterm (<37 weeks)	1.13 (1.08, 1.19)	1.30 (1.20, 1.40)
Gestational age category		
34-36 weeks	1.11 (1.05, 1.17)	1.30 (1.19, 1.42)
32-33 weeks	1.15 (1.01, 1.31)	1.37 (1.11, 1.69)
28-31 weeks	1.20 (1.03, 1.39)	1.26 (0.98, 1.62)
24-27 weeks	1.51 (1.22, 1.88)	1.13 (0.74, 1.74)

© 2024 Ahmed AM et al. *JAMA Network Open*.

eTable 7. Risk Differences (RDs, %) and Ratios (RRs) With 95% CIs for the Association Between PTB and All-Cause Mortality From Birth to 11 Months of Age in the Matched Cohort, Stratified by Sex

**Male sex**		
Category	RD (%)	RR
Preterm (<37 weeks)	2.46 (2.39, 2.54)	11.59 (10.92, 12.29)
**Female sex**		
Category	RD (%)	RR
Preterm (<37 weeks)	2.08 (2.00, 2.16)	11.64 (10.84, 12.49)

© 2024 Ahmed AM et al. *JAMA Network Open*.

eTable 8. Risk Differences (RDs, %) and Ratios (RRs) With 95% CIs for the Association Between PTB and All-Cause Mortality in the Matched Cohort, Stratified by Birth Year

Birth Year	Risk Difference (95% CI)	P-value (interaction)	Risk Ratio (95% CI)	P-value (interaction)	Risk Difference (95% CI)	P-value (interaction)	Risk Ratio (95% CI)	P-value (interaction)
1983	0.63 (0.41, 0.85)	Ref.	1.41 (1.26, 1.57)	Ref.	2.71 (2.47, 2.95)	Ref.	12.10 (10.51, 13.94)	Ref.
1984	0.68 (0.30, 1.06)	0.75	1.47 (1.30, 1.64)	0.62	2.73 (2.40, 3.07)	0.90	11.84 (11.10, 12.71)	0.69
1985	0.75 (0.32, 1.18)	0.43	1.52 (1.39, 1.67)	0.36	2.99 (2.65, 3.35)	0.10	12.98 (11.17, 15.03)	0.58
1986	0.97 (0.32, 1.62)	0.04	1.67 (1.42, 1.96)	0.03	2.81 (2.48, 3.15)	0.55	12.53 (11.05, 14.22)	0.60
1987	0.74 (0.14, 1.34)	0.50	1.54 (1.36, 1.74)	0.25	2.53 (2.19, 2.86)	0.28	12.56 (11.07, 14.20)	0.81
1988	0.61 (0.06, 1.16)	0.89	1.47 (1.29, 1.67)	0.62	2.23 (1.92, 2.55)	0.00	12.63 (11.16, 14.16)	0.27
1989	0.39 (-0.06, 0.84)	0.10	1.32 (1.17, 1.49)	0.43	2.36 (2.05, 2.68)	0.04	12.75 (11.30, 14.31)	0.57
1990	0.43 (-0.10, 0.96)	0.17	1.37 (1.19, 1.56)	0.72	2.50 (2.19, 2.82)	0.21	12.35 (10.89, 13.87)	0.76
1991	0.50 (-0.10, 1.10)	0.35	1.48 (1.31, 1.69)	0.55	2.18 (1.87, 2.60)	0.00	10.97 (9.20, 12.58)	0.72
1992	0.37 (-0.03, 0.77)	0.06	1.36 (1.19, 1.55)	0.66	1.99 (1.67, 2.30)	0.00	14.05 (12.15, 16.11)	0.03
1993	0.45 (0.05, 0.85)	0.18	1.53 (1.29, 1.79)	0.34	1.92 (1.63, 2.22)	0.00	10.97 (9.33, 12.05)	0.67
1994	0.34 (-0.05, 0.73)	0.03	1.44 (1.23, 1.70)	0.79	1.99 (1.70, 2.29)	0.00	12.17 (10.69, 13.77)	0.43
1995	0.46 (0.02, 0.90)	0.20	1.61 (1.41, 1.85)	0.13	1.92 (1.63, 2.22)	0.00	11.10 (9.36, 12.93)	0.85
1996	0.46 (0.02, 0.91)	0.20	1.81 (1.50, 2.21)	0.01	1.65 (1.35, 1.94)	0.00	9.70 (7.71, 12.23)	0.07

© 2024 Ahmed AM et al. *JAMA Network Open*.

eTable 9. Risk Differences (RDs, %) and Ratios (RRs) With 95% CIs for the Association Between PTB and All-Cause Mortality in the Matched Cohort Among a Subsample Linked to Maternal Tax With Matching on Family Income and Rural Residence (1990-1996 Births)

Age interval	Risk Difference, % (95% CI)	Risk Ratio (95% CI)
**1-29 years**	0.37 (0.30, 0.45)	1.48 (1.37, 1.59)
**0-1 year**	1.60 (1.50, 1.70)	10.68 (9.56, 11.91)
**1-5 years**	0.24 (0.19, 0.28)	2.48 (2.15, 2.86)
**6-12 years**	0.39 (0.01, 0.06)	1.54 (1.21, 1.95)
**13-17 years**	0.04 (0.02, 0.07)	1.45 (1.17, 1.79)
**18-29 years**	0.06 (0.00, 0.11)	1.13 (1.01, 1.25)

© 2024 Ahmed AM et al. *JAMA Network Open*.

eFigure 1. Kaplan-Meier Plot of Cumulative Incidence of All-Cause Mortality by Gestational Age Categories in the Unmatched Cohort (Age 1-36 Years)

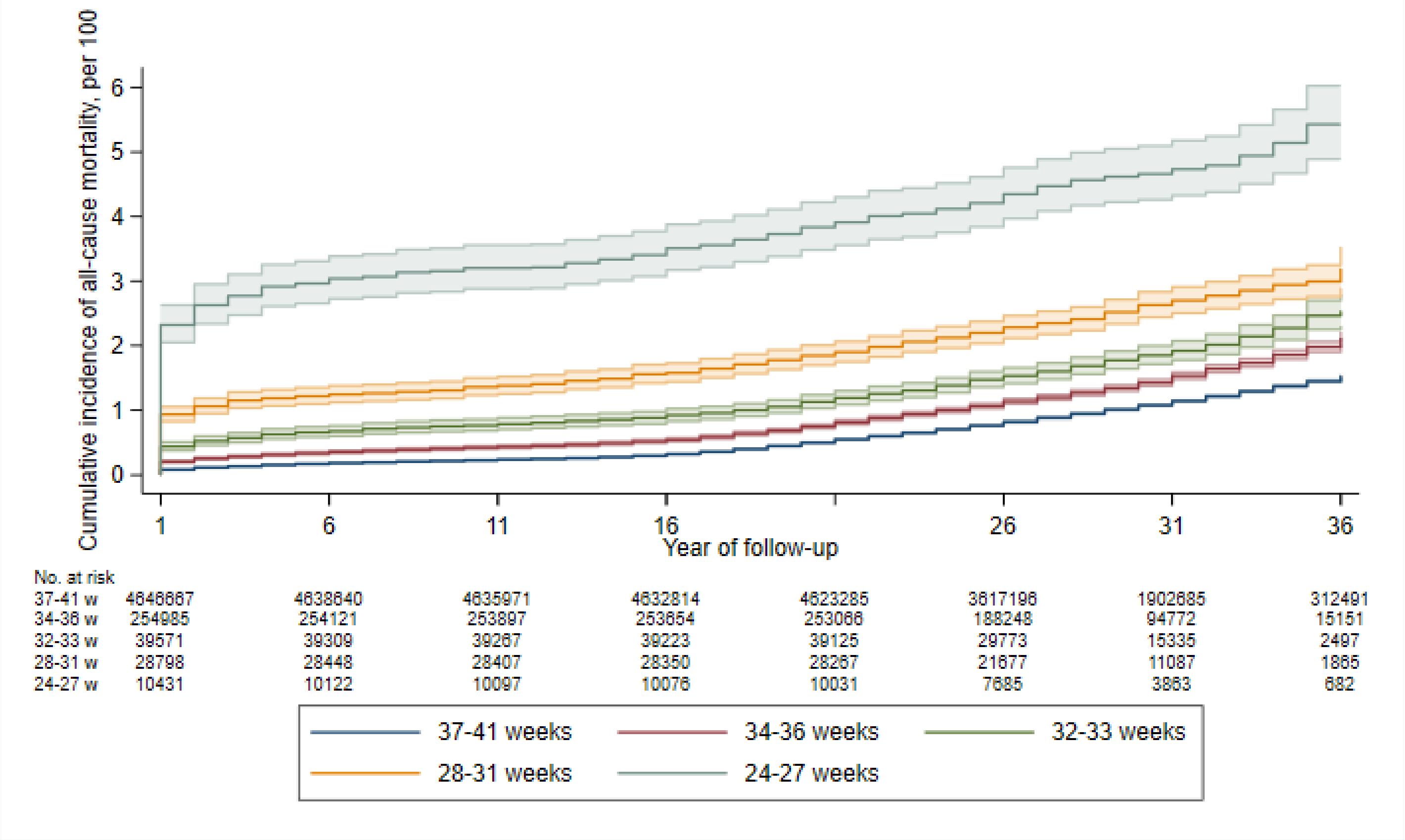

© 2024 Ahmed AM et al. *JAMA Network Open*.

eFigure 2. Kaplan-Meier Plot of Cumulative Incidence of All-Cause Mortality by Preterm Birth in the Matched Cohort (Ages 1-36 Years)

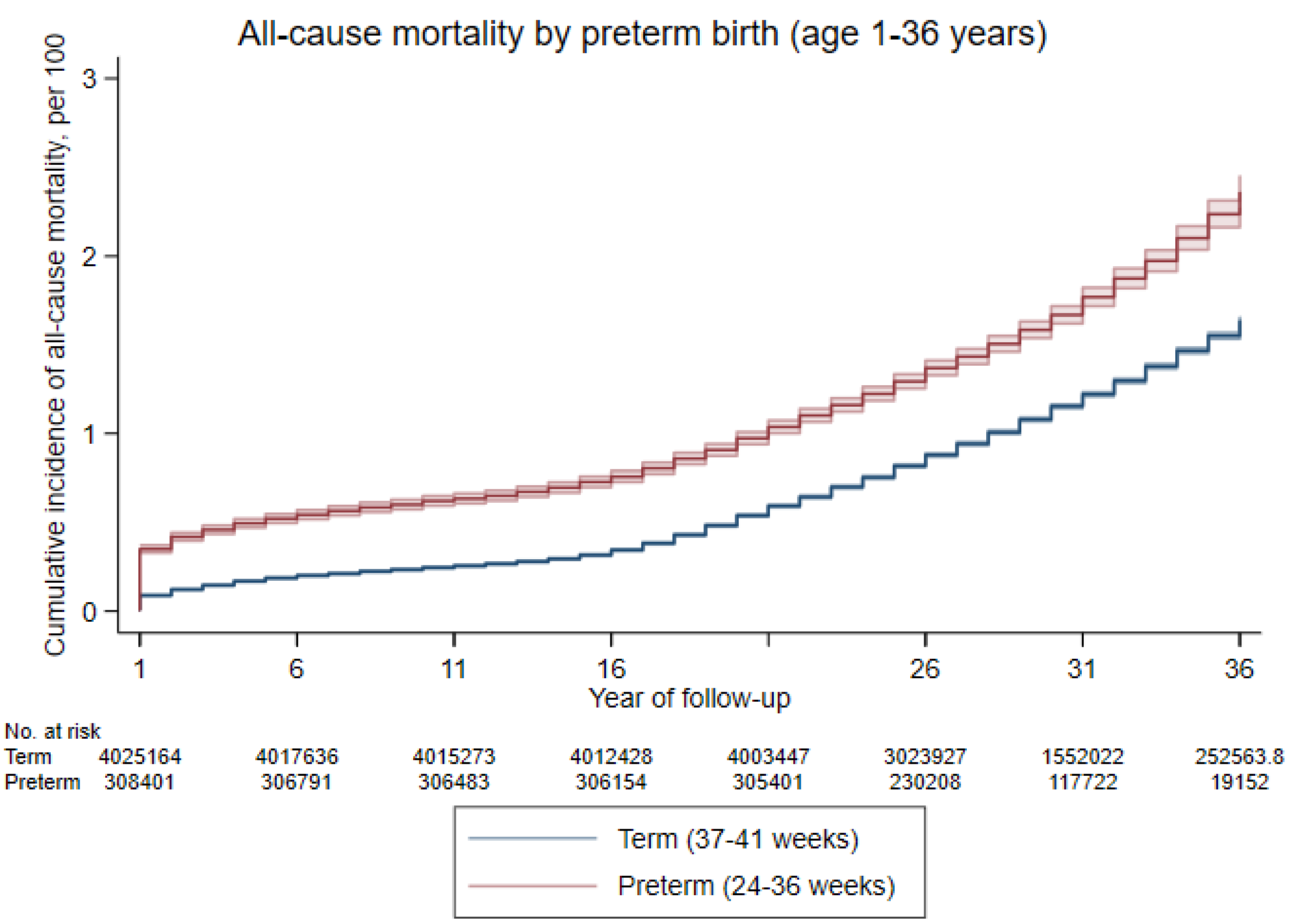

© 2024 Ahmed AM et al. *JAMA Network Open*.

eFigure 3. Risk Differences (RDs, %) and Ratios (RRs) for the Association Between Preterm Birth and All-Cause Mortality in the Matched Cohort, Stratified by Age in Years (Ages 1-36 Years)

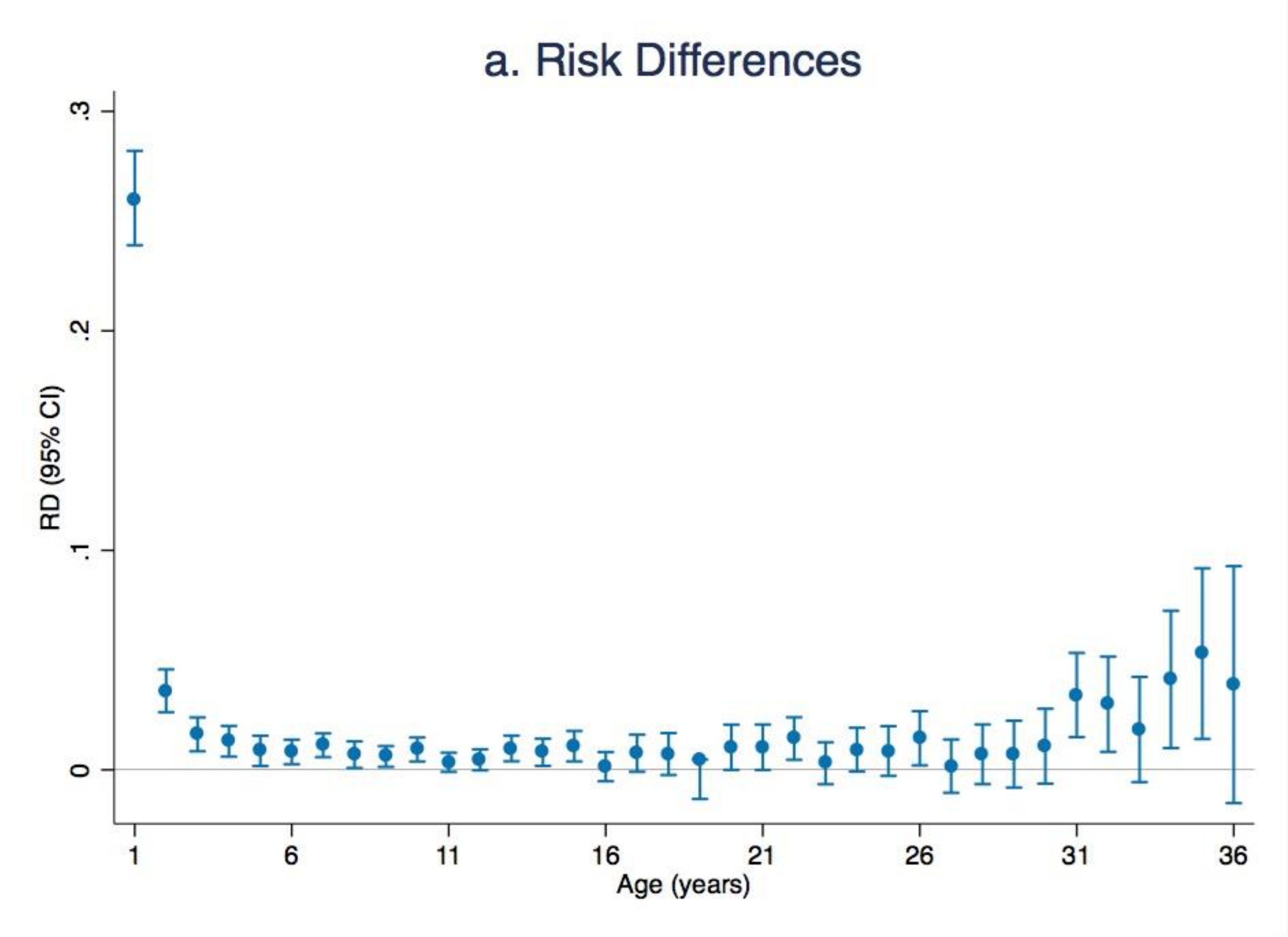

© 2024 Ahmed AM et al. *JAMA Network Open*.

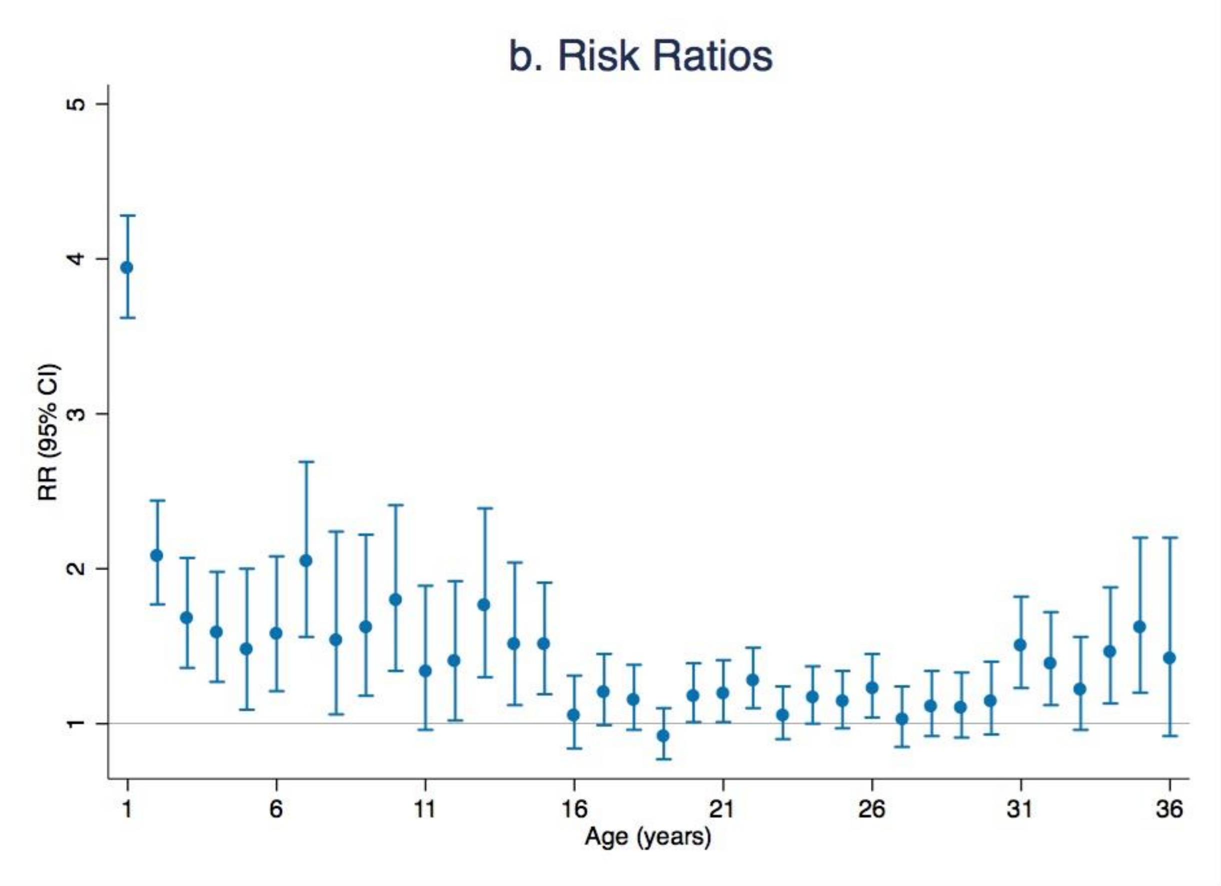

© 2024 Ahmed AM et al. *JAMA Network Open*.

eFigure 4. Kaplan-Meier Plot of Cumulative Incidence of All-Cause Mortality by Preterm Birth in the Matched Cohort (Ages 0-11 Months)

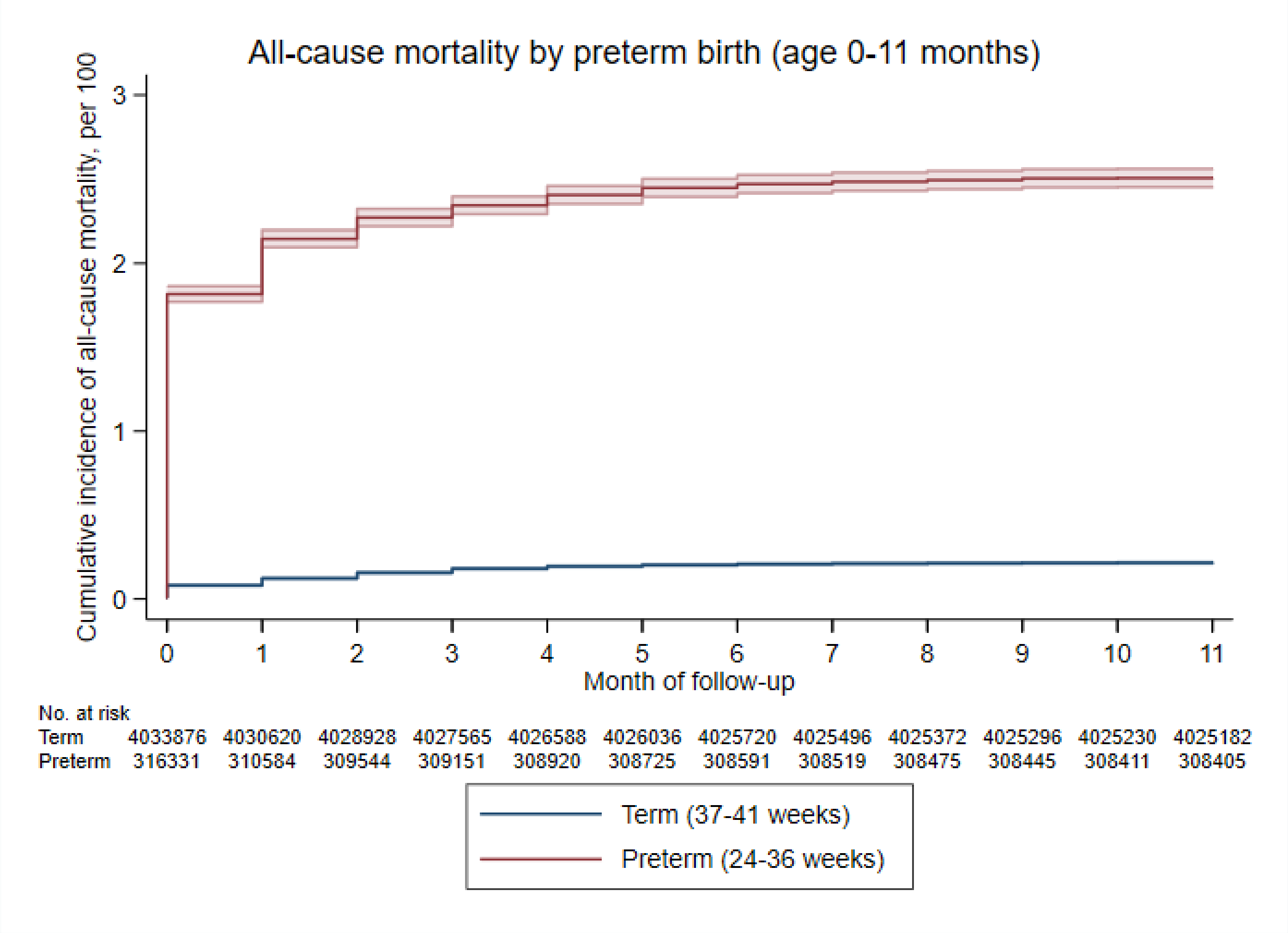

© 2024 Ahmed AM et al. *JAMA Network Open*.

eFigure 5. Risk Differences (RDs, %) and Ratios (RRs) With 95% CIs for the Association Between PTB and All-Cause Mortality in the Matched Cohort, Stratified by Sex

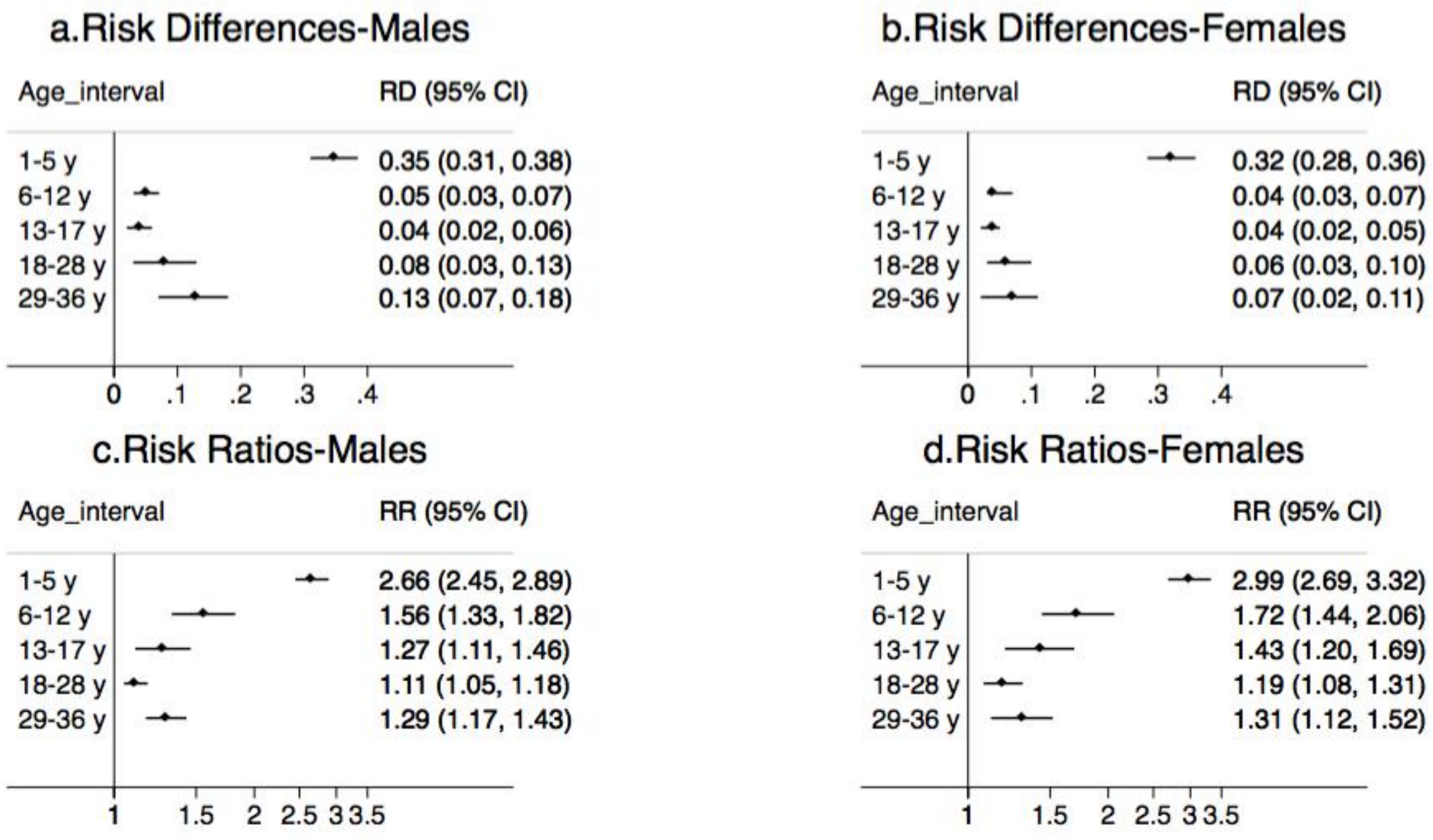

© 2024 Ahmed AM et al. *JAMA Network Open*.
