# Supplementary material for: Short-Term and Long-Term Mortality Risk After Preterm Birth
Source: JAMA Netw Open. 2024 Nov 20;7(11):e2445871. doi: 10.1001/jamanetworkopen.2024.45871 (PMC11579792; doi:10.1001/jamanetworkopen.2024.45871)
Supplement: Supplement 2. — Data Sharing Statement [file jamanetwopen-e2445871-s002.pdf]

Data Sharing Statement

Ahmed. Short-Term and Long-Term Mortality Risk After Preterm Birth. *JAMA Netw Open*. Published November 20, 2024. doi:10.1001/jamanetworkopen.2024.45871

Data
Data available: No
